# Treatment Patterns and Health Outcomes among Patients with HER2 IHC0/-Low Metastatic or Recurrent Breast Cancer

**DOI:** 10.3390/cancers16030518

**Published:** 2024-01-25

**Authors:** Eliya Farah, Chantelle Carbonell, Devon J. Boyne, Darren R. Brenner, Jan-Willem Henning, Daniel Moldaver, Simran Shokar, Winson Y. Cheung

**Affiliations:** 1 Department of Oncology, Cumming School of Medicine, University of Calgary, Calgary, AB T2N 1N4, Canada; 2 Department of Community Health Sciences, University of Calgary, Calgary, AB T2N 1N4, Canada; 3 AstraZeneca Canada Inc., Mississauga, ON L4Y 1M4, Canada

**Keywords:** HER2 negative, HER2 IHC0, HER2-low, metastatic breast cancer, recurrent breast cancer, overall survival

## Abstract

**Simple Summary:**

In clinical practice, human epidermal growth factor receptor (HER2)-negative tumors include triple-negative or luminal breast cancer. HER2-negative breast cancer can be further characterized by immunohistochemical (IHC) and in situ hybridization (ISH) scores into HER2 IHC0 or HER2-low (IHC 1+, 2+/ISH−. Interest in HER2-low tumors has increased as data show the efficacy of HER2-targeted therapies in this subgroup. While several studies have examined treatment variations in the overall breast cancer population, there is limited information on the real-world treatment patterns among patients with HER2 (IHC0/-low) metastatic or recurrent breast cancer. Our study addresses this gap by analyzing treatment and outcomes by HER2 status and found that outcomes were similar in the HER2 IHC0 and -low groups. Our findings add value to the general understanding of the clinical differences between HER2 IHC0/-low and de novo/recurrent disease and have important implications for new effective treatment strategies designed for varying levels of HER2 expression.

**Abstract:**

Improved understanding of the biological heterogeneity of breast cancer (BC) has facilitated the development of more effective and personalized approaches to treatment. This study describes real-world evidence on treatment patterns and outcomes for a population-based cohort of patients with human epidermal growth factor receptor (HER2) IHC0 and -low BC with de novo or recurrent disease from Alberta, Canada. Patients 18+ years old diagnosed with HER2 IHC0/-low, de novo/recurrent BC from 2010 to 2019 were identified using Alberta’s cancer registry. Analyses of these patients’ existing electronic medical records and administrative claims data were conducted to examine patient characteristics, treatment patterns, and survival outcomes. A total of 3413 patients were included in the study, of which 72.10% initiated first line hormonal and non-hormonal systemic therapy. The 1-year overall survival (OS) was 81.09% [95% CI, 79.52–82.69]. Recurrent patients had a higher OS compared to de novo patients: 54.30 months [95% CI, 47.80–61.90] vs. 31.5 months [95% CI, 28.40–35.90], respectively. Median OS was 43.4 months [95% CI, 40.70–47.10] and 35.80 months [95% CI, 29.00–41.70] among patients with HER2-low and HER2 IHC0 cancer, respectively. The study results provide real-world evidence regarding the clinical outcomes of HER2 IHC0/-low and de novo/recurrent disease.

## 1. Introduction

Breast cancer (BC) is considered one of the leading causes of cancer-related morbidity and mortality worldwide [1]. In 2022, approximately 28,600 Canadian women were diagnosed with BC, and 5500 died from the disease [2]. This represents 25% and 14% of all new cancer cases and deaths among Canadian women, respectively.

BC malignancies include a spectrum of intrinsic molecular subtypes with distinct and heterogenous pathological characteristics that respond differently to treatment modalities [3]. This biological heterogeneity is a challenge for clinicians to precisely classify tumors and select therapies that maximize clinical outcomes. Subtypes are classified based on the level of gene expression of certain receptors, including estrogen receptor (ER), progesterone receptor (PR), and human epidermal growth factor receptor (HER2); each differs in risk factors, disease management, prognosis, recurrence rates, and clinical outcomes [4].

HER2-targeted therapies have revolutionized the treatment landscape of patients with HER2-positive (immunohistochemical (IHC) 3+/or IHC2+ and in situ hybridization (ISH) positive score) BC [5]; therefore, testing for HER2 overexpression is considered standard practice during the diagnosis and workup of new patients [6]. Today, clinical applications are moving beyond the dichotomous classification of HER2-positive versus HER2-negative (IHC 0 or IHC 1+, or IHC 2+ and ISH-negative) to the identification of the HER2-low subtype (IHC 1+ or IHC 2+ and ISH-negative), for which new treatment strategies have been granted regulatory approval in Canada, the United States, and Europe, with other treatments currently under active evaluation [7,8,9,10].

Contemporary evidence has suggested that patients with HER2-low tumors may exhibit clinical indicators that align more with patients with HER2-positive disease and can benefit from next-generation HER2-targeted antibody drug conjugates [11]. This was demonstrated in a phase III randomized controlled trial (RCT) (DESTINY-BREAST 04), where trastuzumab deruxtecan (T-DXd) versus the investigator’s choice of chemotherapy improved median progression-free survival (PFS) by 4.8 months and median overall survival (OS) by 6.6 months in patients with HER2-low unresectable and/or metastatic breast cancer (MBC) [12]. Another ongoing phase III RCT is evaluating the efficacy, safety, and tolerability of T-Dxd compared with chemotherapy in patients with HER2-low, hormone receptor (HR)-positive metastatic BC whose disease has progressed on endocrine therapy (DESTINY-BREAST 06) [13]. A similar phase II RCT is assessing the safety, efficacy, pharmacokinetics, and immunogenicity of MRG002, an antibody–drug conjugate, in patients with HER2-low locally advanced or MBC [14].

While several studies have examined treatment variations in the overall BC population, there is limited information on the real-world treatment patterns among patients with HER2 (IHC0/-low) metastatic or recurrent BC. This investigation aims to describe the treatment patterns and corresponding outcomes of women diagnosed with metastatic or recurrent BC in Alberta, Canada, between 2010 and 2019, stratified by HER2 status (IHC0/-low).

## 2. Materials and Methods

### 2.1. Data Sources and Study Design

We conducted a retrospective longitudinal cohort study of individuals aged 18+ years diagnosed with metastatic or recurrent HER2 IHC0 or HER2-low BC between 2010 and 2019 in Alberta, Canada using linked administrative data (Supplementary Figure S1). The study was approved by the Health Research Ethics Board of Alberta, and it was performed in accordance with the provisions of the Declaration of Helsinki and the Good Clinical Practice guidelines as defined by the International Conference on Harmonization. The details of this database have been previously described in Cuthbert et al. [15]. Briefly, individuals diagnosed with cancer were identified using the Alberta Cancer Registry. Demographic and clinical characteristics, as well as the date of death or last known contact with the healthcare system, were abstracted from the registry. Treatment information was obtained from electronic medical records. Notably, no exclusions were made on the basis of the sites of metastasis. Since the provincial cancer registry only captures information at the time of initial diagnosis, an administrative data algorithm was used to identify individuals with recurrent disease.

### 2.2. Treatment and Outcome Definition

Developed in consultation with a practicing senior medical oncologist in Alberta and supported by the validation study performed by Xu et al. (2019) [16], the following algorithm was used to define recurrent disease: (a) 2+ cycles of a non-hormonal systemic therapy consistent with a BC diagnosis 1+ years after the date of treatment for the initial disease, (b) receipt of any radiation therapy 1+ years after the date of treatment for initial disease, (c) cause of death listed as “breast cancer”, (d) or a gap of more than 24 months between successive dispensations of any type of hormone therapy. With respect to HER2 classification, HER2 statuses were assessed at the initial diagnosis using gene expression profiling, ISH, and IHC, whereby HER2 IHC0 was defined as IHC0 and HER2-low was defined as having an IHC1+ or IHC2+/ISH-negative score.

The following algorithm was used to classify the chemotherapy regimen and subsequent lines of therapy: (1) The start date of the line of therapy was defined as the earliest date of systemic treatment (hormonal and non-hormonal, excluding radiation therapy and surgery) given on or after the index date (for patients with metastatic disease, the index date was the date of diagnosis; for patients with recurrent disease, the index date was the earliest date of treatment used to flag recurrence in the administrative data algorithm). (2) All systemic agents received within 30 days of the start date were used to define the regimen. (3) The start date of a subsequent line of therapy was defined as the earliest of the following two dates, if available: (a) date on which patient received any systemic agent not specified in step 2 (note: switches in the type of taxane therapy or switches within the same class of hormone therapy, for example, switching from one type of aromatase inhibitor to another, were not flagged as a new line of therapy); or (b) date on which there was a gap equal to or greater than 240 days between successive non-hormonal systemic treatments. (4) All agents received within 30 days of the start date were used to define the regimen of the subsequent line of therapy. (5) Steps 3 and 4 were repeated to assess subsequent lines of therapy.

The end date of each line of therapy was defined as the earliest of the following four dates: (1) start date of the subsequent line of therapy; (2) date of the last agent received within the line of therapy plus 28 days; (3) date of death, or (4) last known date of follow-up.

The following outcomes were examined: (1) OS, defined as the time from the index date until death from any cause; (2) duration of systemic therapy, defined as the end date minus start date in months; (3) time to initial systemic therapy, defined as the time from the index date to initial systemic therapy in months, if applicable; and (4) time to next treatment, defined as the time from the index date until initiation of a subsequent line of systemic therapy or death in months, whichever occurred first.

### 2.3. Covariates

The following variables were controlled for in the multivariable analyses: age at initial diagnosis (continuous in years), age category ((22–40]/…/(80–99]), year of diagnosis (2010/…/2019), number of Charlson comorbidities assessed within the year prior to diagnosis (0/1/2+; assessed using the algorithm described in Quan et al. [17]), location of residence at diagnosis (urban/rural), neighborhood household income (dollars), and proportion of residents in neighborhoods who achieved a high school degree or higher levels of education (%). We also adjusted for whether the patient had received systemic therapy, radiation, or surgery (yes/no), as well as the number of hospitalizations or emergency room visits (0/1/2/3+) between the time of diagnosis and the index date. We also adjusted for ER (negative/positive/missing), PR (negative/positive/missing), cumulative ER/PR statuses (ER+PR+/ER−PR−/single+/missing), number of metastases (1/2/3/4+), location of metastases (bone/lung/hepatic/lymph nodes/brain/adrenals/peritoneum/other). Individuals missing information on one or more confounders were excluded from the analyses.

### 2.4. Statistical Analyses

Since patient demographics and characteristics were collected at the time of initial diagnosis and were not available at the time of recurrence (for individuals who initially presented with early-stage disease but later had evidence of a recurrence), descriptive statistics were only presented for patients with de novo BC. Continuous variables were analyzed and summarized descriptively with mean, standard deviation, and median. Survival curves and median time-to-event were estimated, and the 95% confidence intervals (CI) were generated using the Kaplan–Meier method for OS. Where applicable, log-rank *p*-values were provided. The treatment duration was represented in months by subtracting the end treatment date from the start treatment date. Sensitivity and subgroup analyses were also conducted to examine OS in relation to HER2 and HR status, as well as disease site, line of treatment, baseline, and treatment characteristics.

## 3. Results

We identified a total of 3413 patients diagnosed with HER2 IHC0/-low, de novo/recurrent BC between 2010 and 2019, of which 565 (16.30%) and 2,848 (83.70%) were classified as HER2 IHC0 and -low, respectively. In addition, 965 (29.30%) and 2448 (71.70%) were considered de novo and recurrent, respectively. The average age at diagnosis for patients with de novo BC was 64 years, 70.40% were HR-positive (ER+ and/or PR+), and 54.50% had only one site of metastasis. The most common metastatic sites were bone (68.50%) and lung (34.10%), followed by lymph nodes (26.30%) and liver (22.40%). The majority, 68.20%, were treated at an academic institution (Table 1). When comparing the HR+ vs. HR− groups, patients with HR+ tumors were older and they had more comorbidities, but fewer metastatic sites (all *p* < 0.05).

Among all patients diagnosed (de novo and recurrent) from 2010 to 2019, 72.10% received first-line (1L) therapy. Among those who initiated a 1L therapy, 49.10% initiated a second-line (2L) therapy; of those who initiated a 2L therapy, 56.50% initiated a third-line (3L) therapy; and of those who initiated a 3L therapy, 41.30% initiated a fourth-line (4L) therapy. The median number of lines was 1.00 (interquartile range [IQR]: 0.00, 2.00) for the overall cohort (Table 2). The most common drugs administered in all lines of therapy were homogenous among HER2 IHC0/-low patients: aromatase inhibitor (AI) monotherapy, followed by tamoxifen monotherapy and capecitabine monotherapy (Table 2). When comparing HR+ vs. HR− groups, endocrine treatments, either as a monotherapy or in combination with a CDK4/6 inhibitor, were more prevalent choices in patients with HR+ tumors, whereas chemotherapy was the more predominant option in those with HR- tumors. The duration of systemic therapy declined with subsequent lines of therapy. First-line therapy lasted the longest at 7.54 months (8.62 months and 7.32 months for patients with HER2 IHC0 and HER2-low, respectively), while fifth-line (5L) therapy was reported with the shortest treatment interval at 4.52 months (4.66 months and 4.51 months for patients with HER2 IHC0 and HER2-low, respectively) (Supplementary Table S2). Patients with HER2 IHC0 disease received, on average, prolonged systemic therapy compared to HER2-low. Time to initial systemic therapy (1L) was on average 3.34 months–2.12 months for HER2 IHC0 and 3.67 months for HER2-low (Supplementary Table S3). With respect to time to the next line of therapy, it took 11.25, 10.20, 8.45, and 7.00 months to initiate 2L, 3L, 4L, and 5L therapies, respectively. When stratified by HER2 status, patients with HER2 IHC0 disease had, on average, shorter time to next therapy intervals compared to patients with HER2-low disease (Supplementary Table S4).

The 1-, 2-, and 5-year survival rates among all patients post diagnosis were 72.91% [95% CI, 70.16–75.77], 55.86% [95% CI, 52.76–59.14], and 24.26% [95% CI, 21.22–27.73], respectively. The median OS from diagnosis among all patients was 28.20 months [95% CI, 26.00–30.70]. When comparing the median OS across the lines of therapy, we observed a decline in OS in subsequent lines of therapies starting from 42.20 months [95% CI, 38.50–44.50], 22.70 [95% CI, 20.30–24.80], 15.90 [95% CI, 13.90–18.10], 12.2 [95% CI, 10.60–15.10], and 11.50 [95% CI, 8.00–13.40], in 1L to 5L, respectively (Table 3).

**Figure 1:**
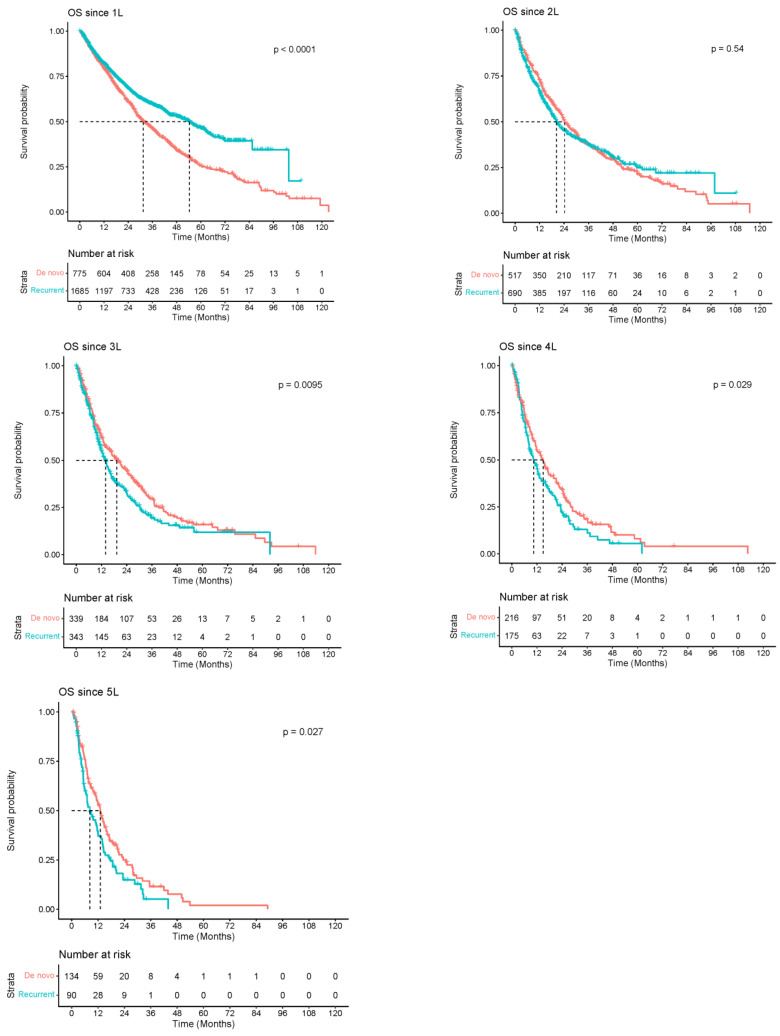
Kaplan–Meier survival curves among patients diagnosed between 2010 and 2019 with de novo or recurrent breast cancer (BC) in Alberta, Canada (*n* = 3413).

In the 1L setting, patients with recurrent BC had a longer median survival of 54.30 months [95% CI, 47.80–61.90] compared to patients with de novo BC, at 31.50 months [95% CI, 28.40–35.90]. However, in subsequent lines of therapy (2L,…, 5L), the median OS was longer among de novo BC compared to recurrent BC (Figure 1).

When considering the impact of HR status on survival, patients with HR-positive disease fared better than those with HR-negative disease. For instance, in the 1L setting, individuals with HER2 IHC0/HR-positive and HER2-low/HR-positive disease had similar median OS values of 41.7 months (95% CI: 34.7–53.7) and 47.4 months (95% CI: 43.7–52.0), respectively. However, individuals with HER2 IHC0/HR-negative and HER2-low/HR-negative disease had consistently worse median OS of 16.0 months (95% CI: 11.1–25.8) and 16.8 months (95% CI: 13.7–22.1), respectively. The OS differences between HR-positive and HR-negative patients were observed across all lines of therapy (Supplementary Table S1).

**Figure 2:**
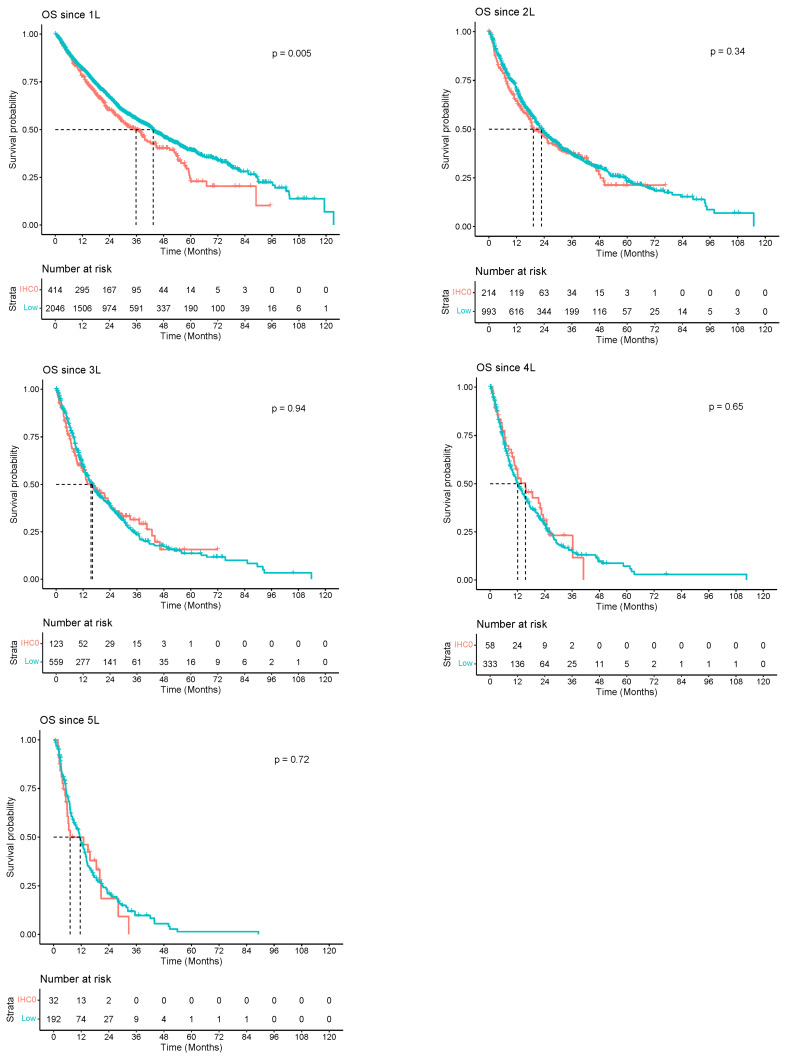
Kaplan–Meier survival curves among patients diagnosed between 2010 and 2019 with HER2 status (IHC0/-low) breast cancer (BC) in Alberta, Canada (*n* = 3413).

When comparing OS by HER2 status (IHC0/-low), median survival was similar in all lines of therapy except for the 1L setting, where patients with HER2-low disease had a slightly better OS compared to patients with HER2 IHC0. In the 1L setting, the median OS in HER2-low was 43.40 months [95% CI, 40.70–47.10] compared to 35.80 months [95% CI, 29.00–41.70] in HER2 IHC0. Median OS declined in subsequent lines of therapy (Figure 2).

## 4. Discussion

We conducted a retrospective cohort study to investigate treatment patterns and outcomes of women diagnosed with metastatic or recurrent BC between 2010 and 2019, stratified by HER2 status (IHC0/low) and de novo/recurrent status in a real-world Canadian setting. We observed that the median OS post diagnosis among all patients in the study cohort was 28.20 months [95% CI, 26.0–30.70]. This finding is consistent with a study by Barcenas C. et al. that found a similar median survival (29.00 months [95% CI, 28.0–30.0]) [18].

Importantly, our results show that patients with HER2 IHC0 and HER2-low BC largely experience similar survival outcomes. A comprehensive examination of the literature, however, demonstrates that survival outcomes between HER2 IHC0/-low tumors have been inconsistent across studies. While we observed relatively similar OS between patients with HER2 IHC0 and HER2-low BC, some studies have reported that patients with HER2-low tumors experience poorer OS compared to those with HER2 IHC0 tumors [19,20,21]. This contrasts other papers that indicate there may be no statistically significant differences in survival between these subgroups [5,22,23,24], which generally align with our current observations. The reasons for these inconsistent findings may be due to differences in study designs, analytical methods, and definitions of HER2 status. Larger, prospective studies are required to confirm the prognostic significance of the varying levels of HER2 expression among patients with BC.

Our analysis also showed that outcomes among patients who initiated 1L therapy were better for recurrent BC than for de novo BC. We suspect that more patients with recurrences had diseases that were amenable to local therapies, such as surgery and radiation therapy, when compared to patients with de novo disease. This likely favored the former group in terms of OS. The location of recurrences may have also played a role [25]. Conversely, in the subsequent 2L+ setting, OS was higher among patients with de novo BC. Several prior studies support this observation [18,26,27,28,29,30]. This survival pattern may be attributed to the exclusion of patients with local recurrences who are less likely to initiate subsequent lines of therapy. The difference in OS may also reflect, in part, variations in baseline characteristics, biological differences, and comorbidities. There was considerable attrition between lines of therapy which limited the precision of these estimates.

Across all lines of therapy in our study, patients with HER2 IHC0/HR-positive and HER2-low/HR-positive BC had similar and better median OS compared to patients with HER2 IHC0/HR-negative and HER2-low/HR-negative BC. This finding is in accordance with recent population-based studies that observed longer disease-free survival (DFS) among patients with HR-positive disease compared to HR-negative disease, regardless of HER2 status (IHC0 or low), and thus suggest HR status to be an independent prognostic factor for DFS in patients with HER2- BC [31,32].

There were strengths and limitations to this investigation. An administrative data-based algorithm was used to identify individuals with recurrent disease and to identify lines of systemic therapy which may have resulted in non-differential misclassification. The classification of HER2 subtypes for recurrent cases was based on measurements of HER2 expression at the time of initial diagnosis. HER2 expression may have changed in recurrent tumors, and this could have impacted survival estimates in patients with recurrent BC. It is known that the location of recurrence is a significant prognostic factor that could impact clinical outcomes. Our algorithm did not distinguish between local vs. distant recurrence, and thus we could not examine the direction and the magnitude of this bias. Another limitation of this investigation was the reliance on administrative data, which do not routinely capture some important clinical covariates that may be of interest, such as performance status, smoking history, disease progression, and low-grade toxicity. Further, our study evaluated data from 2010 to 2019, which limits our findings’ generalizability to more modern treatment practices that have been more recently approved for our study population. For example, BRCA1/2 testing was not considered routine or standard during most of the study timeframe. The main strengths of this study were the generalizability to Alberta and other provinces of Canada. The comprehensive nature of the data used included 3413 patients from 2010 to 2019 across multiple cancer centers in Alberta. This also included the quality of the chemotherapy data, which is routinely captured in electronic medical records, and the short lag period between the current calendar date and the end of follow-up.

## 5. Conclusions

Our study provides new insights into the treatment patterns and outcomes of women diagnosed with HER2 IHC0/-low metastatic and recurrent BC in Alberta, Canada. We examined differences in survival outcomes between HER2 IHC0/-low disease and de novo and recurrent patients with BC. Overall, patients with de novo BC had a better prognosis than patients with recurrent BC, particularly in later lines of treatment. In addition, our study lends support to the notion that patients with HER2-low tumors generally experience similar survival as those with HER2 IHC0 tumors. Our study provides context and offers insights into how emerging therapeutic approaches can be tailored for HER2 IHC0/low, de novo/recurrent BC. Future studies that examine specific subgroups of HER2 IHC0/low patients (such as HR+ vs. HR) in greater detail and research that incorporates multivariable models to determine predictive/prognostic factors would be valuable in guiding more informed treatment decision making.

## Figures and Tables

**Table 1:** Descriptive statistics of patients diagnosed between 2010 and 2019 with de novo breast cancer (BC) in Alberta, Canada (*n* = 965).

Variable	Overall	Not Treated	Treated	*p*-Value
*n*	965	190	775	
Age (mean (SD))	64.01 (14.89)	72.19 (14.13)	62.00 (14.38)	<0.001
Age Category (%)				<0.001
(22, 40]	62 (6.4)	suppressed	suppressed	
(40, 50]	111 (11.5)	suppressed	suppressed	
(50, 60]	204 (21.1)	26 (13.7)	178 (23.0)	
(60, 70]	221 (22.9)	34 (17.9)	187 (24.1)	
(70, 80]	216 (22.4)	45 (23.7)	171 (22.1)	
(80, 99]	151 (15.6)	70 (36.8)	81 (10.5)	
Year of Diagnosis (%)				0.010
2010	73 (7.6)	25 (13.2)	48 (6.2)	
2011	77 (8.0)	18 (9.5)	59 (7.6)	
2012	65 (6.7)	16 (8.4)	49 (6.3)	
2013	90 (9.3)	17 (8.9)	73 (9.4)	
2014	89 (9.2)	20 (10.5)	69 (8.9)	
2015	90 (9.3)	14 (7.4)	76 (9.8)	
2016	125 (13.0)	19 (10.0)	106 (13.7)	
2017	124 (12.8)	23 (12.1)	101 (13.0)	
2018	117 (12.1)	12 (6.3)	105 (13.5)	
2019	115 (11.9)	26 (13.7)	89 (11.5)	
ER Status				<0.001
negative	133 (13.8)	54 (28.4)	79 (10.2)	
positive	830 (86.0)	136 (71.6)	694 (89.5)	
missing	2 (0.2)	0 (0.0)	2 (0.3)	
PR Status				<0.001
negative	278 (28.8)	85 (44.7)	193 (24.9)	
positive	683 (70.8)	105 (55.3)	578 (74.6)	
missing	4 (0.4)	0 (0.0)	4 (0.5)	
ER/PR Status				<0.001
ER− PR−	129 (13.4)	53 (27.9)	76 (9.8)	
ER+ PR+	679 (70.4)	104 (54.7)	575 (74.2)	
Single+	153 (15.9)	33 (17.4)	120 (15.5)	
missing	4 (0.4)	0 (0.0)	4 (0.5)	
No. of Metastases (%)				0.020
1	507 (52.5)	87 (45.8)	420 (54.2)	
2	232 (24.0)	44 (23.2)	188 (24.3)	
3	127 (13.2)	28 (14.7)	99 (12.8)	
4+	92 (9.5)	28 (14.7)	64 (8.3)	
missing	7 (0.7)	3 (1.6)	4 (0.5)	
Location of Metastases (%)	NA	NA	NA	NA
bone (%)	661 (68.5)	127 (66.8)	534 (68.9)	0.645
lung (%)	329 (34.1)	72 (37.9)	257 (33.2)	0.251
hepatic (%)	216 (22.4)	50 (26.3)	166 (21.4)	0.176
lymph nodes (%)	254 (26.3)	45 (23.7)	209 (27.0)	0.407
brain (%)	26 (2.7)	11 (5.8)	15 (1.9)	0.007
adrenals (%)	30 (3.1)	13 (6.8)	17 (2.2)	0.002
skin (%)	33 (3.4)	suppressed	suppressed	0.999
peritoneum (%)	37 (3.8)	12 (6.3)	25 (3.2)	0.076
other (%)	101 (10.5)	32 (16.8)	69 (8.9)	0.002
Systemic Tx Institution (%)				<0.001
academic	658 (68.2)	0 (0.0)	658 (84.9)	
community	117 (12.1)	0 (0.0)	117 (15.1)	
did not initiate systemic Tx	190 (19.7)	190 (100.0)	0 (0.0)	

BC: breast cancer. ER: estrogen receptor. NA: not available. PR: progesterone receptor. SD: standard deviation. Tx: treatment.

**Table 2:** Lines of therapy administered among all patients diagnosed between 2010 and 2019 with recurrent or de novo breast cancer (BC) in Alberta, Canada (*n* = 3413).

Variable	Overall	Recurrent	De Novo	*p*-Value
*n*	3413	2448	965	
No. lines (median [IQR])	1.00 [0.00, 2.00]	1.00 [0.00, 2.00]	2.00 [1.00, 3.00]	
Initiated 1L (%)	2460 (72.1)	1685 (68.8)	775 (80.3)	<0.001
1L regimens (%)				<0.001
AI mono	900 (36.6)	583 (34.6)	317 (40.9)	
Tamoxifen mono	457 (18.6)	375 (22.3)	82 (10.6)	
Capecitabine mono	265 (10.8)	213 (12.6)	52 (6.7)	
AI + Palbociclib	207 (8.4)	112 (6.6)	95 (12.3)	
Taxane mono	137 (5.6)	62 (3.7)	75 (9.7)	
Taxane + chemo	119 (4.8)	95 (5.6)	24 (3.1)	
Chemo combo	92 (3.7)	71 (4.2)	21 (2.7)	
FEC	78 (3.2)	20 (1.2)	58 (7.5)	
Other chemo mono	60 (2.4)	52 (3.1)	<10	
Other AI combo	42 (1.7)	19 (1.1)	23 (3.0)	
AI + Everolimus	40 (1.6)	36 (2.1)	<10	
Other	32 (1.3)	21 (1.2)	11 (1.4)	
Fulvestrant	18 (0.7)	15 (0.9)	<10	
Targeted mono	13 (0.5)	11 (0.7)	<10	
Initiated 2L (%)	1207 (49.1)	690 (40.9)	517 (66.7)	<0.001
2L Regimens (%)				<0.001
AI mono	287 (23.8)	171 (24.8)	116 (22.4)	
Capecitabine mono	190 (15.7)	113 (16.4)	77 (14.9)	
Tamoxifen mono	154 (12.8)	87 (12.6)	67 (13.0)	
Taxane mono	135 (11.2)	63 (9.1)	72 (13.9)	
Other chemo mono	99 (8.2)	81 (11.7)	18 (3.5)	
AI + Palbociclib	82 (6.8)	26 (3.8)	56 (10.8)	
Chemo combo	62 (5.1)	40 (5.8)	22 (4.3)	
AI + Everolimus	56 (4.6)	27 (3.9)	29 (5.6)	
Other AI combo	56 (4.6)	23 (3.3)	33 (6.4)	
Fulvestrant	37 (3.1)	26 (3.8)	11 (2.1)	
Taxane + chemo	25 (2.1)	19 (2.8)	<10	
Targeted mono	17 (1.4)	<10	<10	
Other	<10	<10	<10	
Initiated 3L (%) 3L	682 (56.5)	343 (49.7)	339 (65.6)	<0.001
Regimens (%)				<0.001
Capecitabine mono	136 (19.9)	59 (17.2)	77 (22.7)	
AI mono	109 (16.0)	54 (15.7)	55 (16.2)	
Taxane mono	76 (11.1)	39 (11.4)	37 (10.9)	
Tamoxifen mono	60 (8.8)	25 (7.3)	35 (10.3)	
Other chemo mono	58 (8.5)	33 (9.6)	25 (7.4)	
Eribulin mono	48 (7.0)	42 (12.2)	<10	
Chemo combo	47 (6.9)	25 (7.3)	22 (6.5)	
Fulvestrant	43 (6.3)	17 (5.0)	26 (7.7)	
Other AI combo	34 (5.0)	19 (5.5)	15 (4.4)	
AI + Evrolimus	26 (3.8)	<10	18 (5.3)	
AI + Palbociclib	26 (3.8)	<10	18 (5.3)	
Other	19 (2.8)	14 (4.1)	<10	
Initiated 4L (%)	391 (57.3)	175 (51.0)	216 (63.7)	0.001
4L regimens (%)				0.036
AI mono or combo	79 (20.2)	28 (16.0)	51 (23.6)	
Taxane mono	66 (16.9)	34 (19.4)	32 (14.8)	
Other chemo mono	60 (15.3)	30 (17.1)	30 (13.9)	
Capecitabine mono	51 (13.0)	17 (9.7)	34 (15.7)	
Eribulin mono	43 (11.0)	26 (14.9)	17 (7.9)	
Chemo combo	32 (8.2)	18 (10.3)	14 (6.5)	
Other	31 (7.9)	11 (6.3)	20 (9.3)	
Fulvestrant	29 (7.4)	11 (6.3)	18 (8.3)	
Initiated 5L (%)	224 (57.3)	90 (51.4)	134 (62.0)	0.045

AI: aromatase inhibitor. 1L: first line. 2L: second line. 3L: third line. 4L: fourth line. 5L: fifth line. FEC: 5-fluorouracil, epirubicin, and cyclophosphamide. IQR: interquartile range.

**Table 3:** Median overall survival (months) and overall survival (proportion), by lines of therapy administered among all patients diagnosed between 2010 and 2019 with HER2 IHC0/-low metastatic or recurrent breast cancer (BC) in Alberta, Canada (*n* = 3413).

Time Zero	Median Overall Survival, Months (95% CI)	1-Year Overall Survival (95% CI)	2-Year Overall Survival (95% CI)	5-Year Overall Survival (95% CI)
diagnosis	28.2 (26.0–30.7)	0.7291 (0.7016–0.7577)	0.5586 (0.5276–0.5914)	0.2426 (0.2122–0.2773)
1L	42.2 (38.5–44.5)	0.8109 (0.7952–0.8269)	0.6568 (0.6369–0.6773)	0.3715 (0.3440–0.4013)
2L	22.7 (20.3–24.8)	0.6945 (0.6682–0.7219)	0.4770 (0.4472–0.5088)	0.2274 (0.1950–0.2652)
3L	15.9 (13.9–18.1)	0.5875 (0.5500–0.6276)	0.3891 (0.3502–0.4322)	0.1399 (0.1048–0.1867)
4L	12.2 (10.6–15.1)	0.5102 (0.4603–0.5655)	0.2902 (0.2434–0.3461)	0.0679 (0.0364–0.1267)
5L	11.5 (8.8–13.4)	0.4735 (0.4086–0.5488)	0.2094 (0.1559–0.2812)	0.0127 (0.0019–0.0847)

CI: Confidence intervals.

## Data Availability

Data are available through a request to the Alberta Cancer Registry.

## Supplementary Materials

The following supporting information can be downloaded at: https://www.mdpi.com/article/10.3390/cancers16030518/s1, Figure S1: Schematic diagram to provide a visual representation of the study background, methodology, and key findings; Table S1: Median survival (months), stratified by HER2 IHC0/low and HR status; Table S2: Duration of therapy (months); Table S3: Time from diagnosis to initiation of 1L systemic therapy (weeks); Table S4: Time to next treatment (months).
