# Supplementary material for: Treatment Patterns and Health Outcomes among Patients with HER2 IHC0/-Low Metastatic or Recurrent Breast Cancer
Source: Cancers (Basel). 2024 Jan 25;16(3):518. doi: 10.3390/cancers16030518 (PMC10854846; doi:10.3390/cancers16030518)
Supplement: Supplementary file 1 [file cancers-16-00518-s001.zip › cancers-2769455-supplementary.pdf]

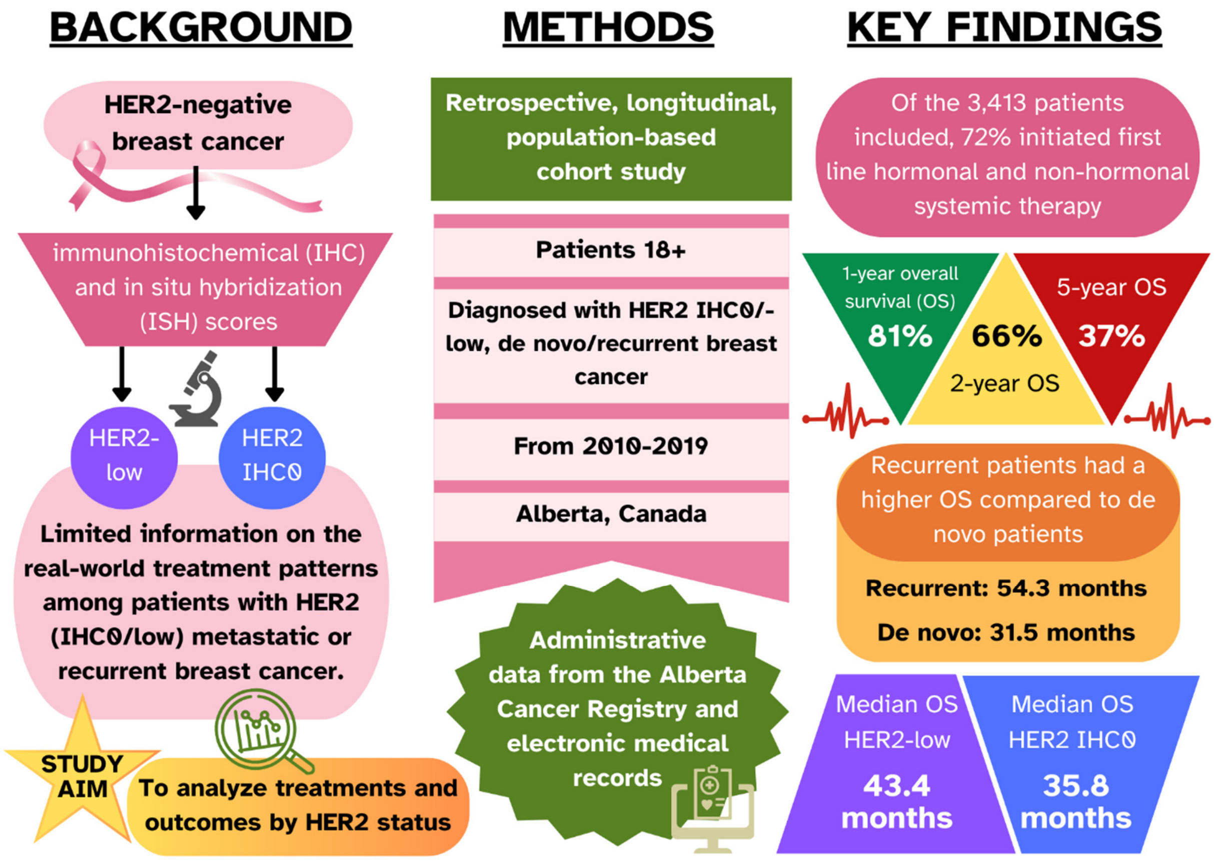

Figure S1: Schematic diagram to provide a visual representation of the study background, methodology, and key findings.

Supplemental Table S1. Median survival (months), stratified by HER2 IHC0/low and HR status.

Time zero	Strata	Median	Lower 95% CI	Upper 95% CI
1L	HER2 Low & HR-	16.8	13.7	22.1
1L	HER2 Low & HR+	47.4	43.7	52.0
1L	HER2 IHC0 & HR-	16.0	11.1	25.8
1L	HER2 IHC0 & HR+	41.7	34.7	53.7
2L	HER2 Low & HR-	13.0	11.7	16.2
2L	HER2 Low & HR+	25.2	23.0	29.5
2L	HER2 IHC0 & HR-	11.0	8.3	21.4
2L	HER2 IHC0 & HR+	25.1	18.5	43.7
3L	HER2 Low & HR-	10.1	7.9	12.9
3L	HER2 Low & HR+	17.3	15.2	21.3
3L	HER2 IHC0 & HR-	7.7	4.7	14.0
3L	HER2 IHC0 & HR+	23.0	13.1	40.5
4L	HER2 Low & HR-	5.1	4.4	9.1
4L	HER2 Low & HR+	14.1	11.8	17.2
4L	HER2 IHC0 & HR-	8.1	3.7	NA
4L	HER2 IHC0 & HR+	22.0	10.6	NA

1L: first line. 2L: second line. 3L: third line. 4L: fourth line. CI: Confidence intervals; HER2: human epidermal growth factor receptor; NA: not applicable

Supplemental Table S2. Duration of therapy (months).

line	n	KM	median	p25	p75	mean	sd
1L	2460	3.88	3.68	0.92	9.85	7.54	9.69
2L	1207	3.91	3.48	1.58	8.65	6.95	8.89
3L	682	4.37	3.96	1.61	7.99	6.67	7.77
4L	391	3.45	3.22	1.84	6.44	5.18	5.63
5L	224	3.19	2.94	1.50	5.80	4.53	4.28
HER2 IHC0							
1L	414	4.83	4.60	1.81	11.28	8.62	10.42
2L	214	3.68	3.39	1.77	7.36	6.50	7.99
3L	123	4.60	4.34	1.61	8.09	6.96	8.65
4L	58	3.88	3.45	1.85	6.50	5.37	5.50
5L	32	2.60	2.56	1.81	6.79	4.66	4.33
HER2-Low							
1L	2046	3.68	3.48	0.92	9.43	7.32	9.53
2L	993	4.04	3.65	1.45	8.71	7.05	9.07
3L	559	4.27	3.88	1.61	7.99	6.60	7.57
4L	333	3.45	3.12	1.84	6.44	5.15	5.66
5L	192	3.22	3.14	1.44	5.79	4.51	4.28

HER2: human epidermal growth factor receptor; KM: Kaplan-Meier; sd: standard deviation

Supplemental Table S3. Time from diagnosis to initiation of 1L systemic therapy (weeks).

Tx	n	med	p25	p75	mean	sd
All Patients	775	6.00	3.86	11.14	14.51	30.59
HER2-Null	167	5.00	3.43	8.00	9.22	18.79
HER2-Low	608	6.29	3.86	11.61	15.96	32.96

HER2: human epidermal growth factor receptor; sd: standard deviation; Tx: treatment

Supplemental Table S4. Time to next treatment (months).

line	n	med	p25	p75	mean	sd
1L	1207	7.10	3.17	15.42	11.25	11.26
2L	682	6.28	3.25	13.12	10.20	9.94
3L	391	5.75	2.86	10.31	8.35	8.37
4L	224	4.41	2.79	8.12	7.00	7.63
HER2 IHC0						
1L	214	6.97	3.01	14.59	10.84	11.64
2L	123	5.16	3.12	11.06	8.93	9.23
3L	58	5.92	3.00	11.12	8.84	8.91
4L	32	5.29	2.99	6.82	5.61	3.34
HER2-Low						
1L	993	7.13	3.19	15.88	11.34	11.18
2L	559	6.64	3.40	13.27	10.47	10.07
3L	333	5.72	2.83	10.03	8.26	8.28
4L	192	4.37	2.79	8.52	7.24	8.11

1L: first line. 2L: second line. 3L: third line. 4L: fourth line; HER2: human epidermal growth factor receptor; sd: standard deviation
